# Assessment of the effect of iris colour and having children on 5-year risk of death after diagnosis of uveal melanoma: a follow-up study

**DOI:** 10.1186/1471-2415-14-42

**Published:** 2014-04-01

**Authors:** Andrea Schmidt-Pokrzywniak, Sven Kalbitz, Oliver Kuss, Karl-Heinz Jöckel, Norbert Bornfeld, Andreas Stang

**Affiliations:** 1 Institute of Clinical Epidemiology, Medical Faculty, University of Halle-Wittenberg, Magdeburger Str 8, 06097 Halle, Germany; 2 Institute of Medical Epidemiology, Biostatistics, and Informatics, Medical Faculty, University of Halle-Wittenberg, Magdeburger Str 8, 06097 Halle, Germany; 3 Institute of Medical Informatics, Biometry and Epidemiology, University Hospital, University of Duisburg-Essen, Hufelandstr 55, 45122 Essen, Germany; 4 Division of Ophthalmology, University Hospital, University of Duisburg-Essen, Hufelandstr 55, 45122 Essen, Germany; 5 School of Public Health, Boston University, 715 Albany Street, Boston, MA 02118, USA

**Keywords:** Uveal melanoma, Prognostic factors, Follow-up study, Iris colour, Children

## Abstract

**Background:**

To examine the all-cause mortality and uveal melanoma specific mortality among newly diagnosed uveal melanoma patients after five years. Furthermore, we assess of the effect of iris colour and having children on 5-year risk of death after diagnosis of uveal melanoma. Therefore, we assess the performance of an individual prediction model of survival from uveal melanoma.

**Methods:**

A cohort of 459 patients aged 45 to 79 years with newly diagnosed uveal melanoma was recruited between 2002 and 2004 from the Division of Ophthalmology, University of Essen, Germany. Survival probabilities were estimated by Kaplan-Meier survival analysis. The clinical and histopathological characteristics were obtained from medical records. Iris colour and childbearing history were assessed at baseline by a computer-assisted telephone interview. We used crude and multivariable Cox proportional hazards regression to estimate unadjusted and adjusted hazard ratios (HR) and corresponding 95% confidence intervals (95%CIs) with respect to death from uveal melanoma and death from all causes. We used the Cox model to estimate adjusted probabilities of primary events. For computing Harrell’s C statistics, we used a Cox model including the prognostics factors gender, age at diagnosis, ciliary body involvement, largest basal tumour diameter, and iris colour.

**Results:**

The 5-year uveal melanoma-specific survival probability was 82.9% (95% CI: 79.1-86.3). Main prognostic factors for the death of uveal melanoma were ciliary body involvement (HR: 1.7 (95% CI:1.0-2.8)), largest basal tumour diameter >15 mm HR: 7.0 (95% CI: 3.5-13.9), light iris colour (HR: 2.3 (95% CI: 0.9-5.8), having children (HR: 0.6 (95% CI: 0.2 - 1.7)), and gender (HR: 0.7 (95% CI: 0.4-1.1)). The value of the bootstrap-corrected C statistics was 0.76 (95% CI: 0.74-0.77).

**Conclusion:**

Beyond the established prognostic factors, light iris colour also appears to be a prognostic factor for death from uveal melanoma.

## Background

Although a rare disease, uveal melanoma of the eye is the most common primary intraocular malignancy in adults, with an incidence rate of up to 8 per 1,000,000 person years (age-standardised, world standard) in Europe [1,2] and the fatality rate is high despite modern treatment modalities. Bergman et al. reported an observed survival after 5 and 10 years of 60% and 43%, respectively. The relative survival, taken as the estimate of the probability of death due to uveal melanoma was 70% after 5 years (68% in men and 72% in women) and 59% after 10 years (58% in men and 61% in women), respectively [3]. In the European Cancer Registry (EUROCARE)–based study of survival and care of patients with cancer, which included data from 67 cancer registries with a combined population of 100 million persons in 22 European countries, the 5-year relative uveal melanoma survival ranged from 63% to 71% [4]. Patients are at risk of developing metastases up to 20 years after the initial diagnosis. The most common site for metastatic uveal melanoma is the liver. 80% of metastatic patients die within one year and 92% within 2 years of the diagnosis of metastases [5].

Uveal melanomas affect both sexes at equal rates, but the reported disease-specific mortality is higher among men [4,6]. The risk of death was 10% higher in males than in female patients and was about 2 and 2.5 times higher, respectively, in the groups aged 55 to 64 years and 75 years or older compared with the group aged 54 years or younger [4]. An important predictor of time to death is the largest basal tumour diameter. In the Collaborative Ocular Melanoma Study (COMS), Hawkins et al. found cumulative age adjusted rates of death from any cause at 5 years after diagnosis of 49% with a maximum basal tumour diameter of ≥18 mm and of 31% with a diameter of <18 mm, respectively [7]. Further prognostic factors include the involvement of the ciliary body, extrascleral extension, and an inflammatory phenotype [8]. Specific genetic alterations that are associated with the development of metastases have been identified, such as monosomy [8,9]. Several studies have shown that monosomy 3 is also an important prognostic factor with regard to predicting death due to metastatic disease [8-10]. Damato et al. reported that the best predictive index can be obtained by using several parameters, including monosomy 3, basal tumour diameter, and epithelioid cellularity, creating a combined prognostic index [10]. Several studies showed that light iris colour is one of the established risk factors for uveal melanoma, [11-13] but only one study has examined the association between light iris colour and the risk of death from uveal melanoma [14]. Regan et al. found that patients with blue or grey irises had a 2-fold increased risk of metastatic death from uveal melanoma, independent of other risk factors, compared to those with darker irises [14]. Another study showed that overall adjusted death rates from metastasis were approximately 25% higher in nulliparous women than in women who had given birth. The protective influence of parity was strongest in the early period following diagnosis and treatment. The level of protection increased with the number of live births [15].

Today, the majority of patients are treated by eye-preserving therapies (63%) [5,16], which mainly involve radiation brachytherapy and teletherapy. Estimates of prognosis among these patients can be based only on clinical factors. Cook et al. found that the majority of patients want to know their prognosis for survival, even when they are told that prognostication is unlikely to improve their chances of prolonging life [17]. The patients who are most distressed are those who cannot be given a prognosis because genetic testing has failed [18].Therefore it is necessary to identify further prognostic factors which are relevant for death from uveal melanoma that are not based on histological and/or genetics factors. The purpose of our study was to investigate the all-cause mortality and uveal melanoma specific mortality among newly diagnosed uveal melanoma patients after five years. Furthermore, we assessed the association between iris colour and having children on the risk of death from uveal melanoma. In addition, we assess the individual prediction performance for survival of uveal melanoma death. For this we computed C statistics.

## Methods

### Patients

The analysis was based on a cohort of 459 patients aged 25 to 79 years with a diagnosis of uveal melanoma between 2002 and 2004. The baseline case recruitment was hospital-based and took place in the Division of Ophthalmology, University of Essen, which is a referral centre for eye cancer in Germany. Cases were defined as patients with uveal melanoma as identified by the *International Classification of Diseases, Tenth Revision* (*ICD-10*) as C69.3 (Choroid) or C69.4 (Ciliary body) and by *International Classification of Diseases for Oncology* morphology codes 8720/3 to 8774/3 (uveal melanoma) [19]. To evaluate vital status during follow-up we sent a questionnaire to all 459 patients after a median follow-up time of 58.4 months. After repeated mailings to non-respondents, we received answers from 77% of the patients. For the remaining non-respondents we evaluated the vital status via registration residents’ offices.

The study was approved by the ethics committee of the Medical Faculty in Essen, Germany (01-113-1713).

Informed consent was obtained from all responding patients.

### Exposure assessment and outcome

The clinical and histopathological characteristics were obtained from medical records. Iris colour and childbearing history were assessed at baseline by a computer-assisted telephone interview (CATI) [19]. The CATI contained a question about eye colour with the following categorical answers: blue, grey, green, hazel, brown, and black. Furthermore, women were asked: how many children do you have (including already deceased children)? The information on cause of death was based on official death certificates in 89%, statements from patients’ ophthalmologists and/or general physicians in 2%, and on statements from relatives or friends in 9%. The statements were retrieved by personal interviews.

### Statistical analysis

Survival probabilities were estimated by Kaplan-Meier survival analysis. We used crude and multivariable Cox proportional hazards regression to estimate unadjusted and adjusted hazard ratios (HR) and corresponding 95% confidence intervals (95%CIs) with respect to death from uveal melanoma and death from all causes. We checked the assumption of proportional hazards by use of Schoenfeld residual plots. We used the Cox model to estimate adjusted probabilities of primary events based on mean values of the covariates in the model. We identified minimally sufficient adjustment sets depending on the outcome using causal diagrams that represented the presumed associations between exposure, outcome, and other variables [20]. For computing Harrell’s C statistics, we used a Cox model including the prognostic factors gender, age at diagnosis, ciliary body involvement, largest basal tumour diameter, and iris colour as covariates and corrected for over-optimism by bootstrapping (1000 runs) [21]. The over-optimism-corrected C statistics is given with a non-parametric 95% bootstrap confidence interval. A value of 0.5 indicates that the model is no better than chance at predicting the outcome, while a value of 1.0 indicates that the model perfectly identifies the outcome. Models are typically considered reasonable when the C-statistic is higher than 0.7 and strong when C exceeds 0.8 [21]. All analyses were performed using the statistical software SAS Version 9.2 [22]. This study was conducted in accordance with the German guidelines of Good Epidemiological Practice [23].

## Results

Patient baseline characteristics are given in Table 1.

**Table 1:** Baseline characteristics of incidence uveal melanoma patients; treated in Germany, Essen 2002-2004

	**All**		**Women**		**Men**	
	**N**	**%**	**N**	**%**	**N**	**%**
	459	100	216	47	243	53
**Age groups (years)**						
20-49	90	19..6	46	21.2	44	18.1
50-59	111	24.2	56	26.0	55	25.5
60-69	192	41.8	83	38.4	109	44.8
70-75	66	14.4	31	14.4	35	14.4
**Tumour stage**						
T1	84	18.3	39	18.0	45	18.5
T2	155	33.7	82	37.9	73	30.0
T3	198	43.1	87	40.3	111	45.8
T4	7	1.5	2	0.9	5	2.0
Missing	15	3.4	6	2.9	9	3.7
**Largest basal tumour diameter (mm)**						
≤ 10	168	37.6	78	36.1	90	37.0
> 10 - ≤15	197	42.9	104	48.1	93	38.3
> 15	79	17.2	28	13.0	51	21.0
Missing	15	3.3	6	2.8	9	3.7
**Extra ocular growth**						
No	451	98.3	2	0.9	5	2.1
Yes	7	1.5	2	0.9	5	2.1
**Ciliary body involvement**						
No	368	80.2	172	80.0	196	81.7
Yes	87	19.0	43	20.0	44	18.3
Missing	4	0.9	1	0.5	3	
**Iris colour**						
Light^1)^	397	86.5	190	88.0	207	85.2
Dark^2)^	62	13.5	26	12.0	36	14.8
**Number of children**						
0			28	12.9		
1			62	28.7		
2			87	40.3		
≥3			39	18.1		

^1)^grey, blue, green, hazel ^2)^brown, black.

In 79% of the 459 cases only the choroid was involved, in 1% only the ciliary body, in 2% the iris, and in 18% the ciliary body and other parts of the uvea were involved. With regard to primary therapy, 12% of the cases underwent enucleation, 67% were treated by brachytherapy, 9% by proton beam radiotherapy, 6% by stereotactic radiotherapy, 2% by transpupillary thermotherapy, and 4% by other eye-preserving therapies. Among women, 51% of tumours were diagnosed in the left eye, while this was 56% in men.

### Follow-up data

The median follow-up time was 58 months. Follow-up data were available from 457 (99.5%) patients; two patients were lost to follow-up. During the follow-up period, 95 patients (22%) died, of whom 79 (83%) died due to metastatic melanoma and 11 died from other causes (12%). In 5 patients (5%), the cause of death is unknown.

### Survival analysis

**Figure 1:**
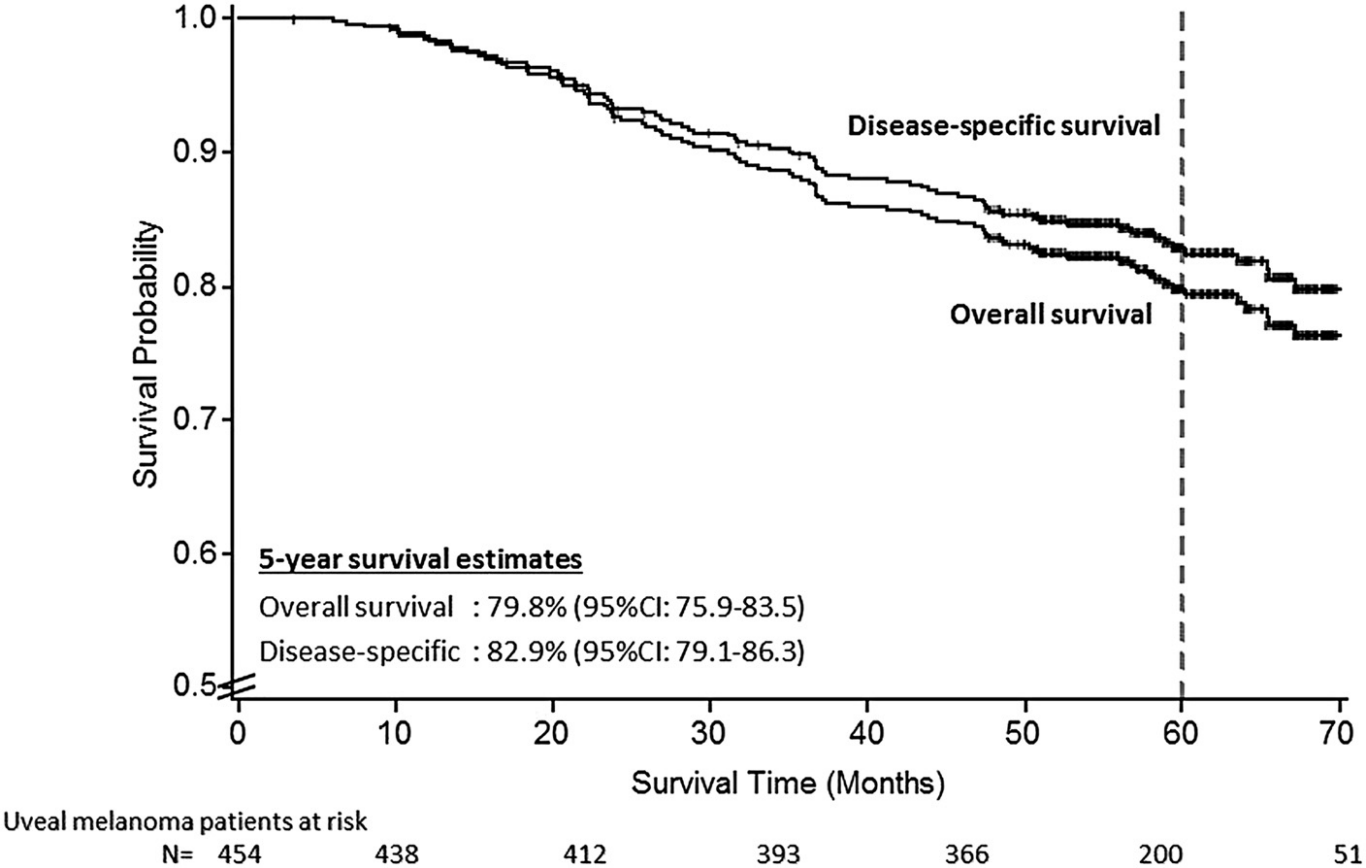
Survival probabilities for overall and disease-specific survival among 459 uveal melanoma patients, Germany, 2002–2010.

Kaplan–Meier analysis revealed a 5-year overall survival probability of 79.8% (95% CI: 75.9-83.5), and uveal melanoma-specific survival of 82.9% (95% CI: 79.1-86.3), (Figure 1).

**Figure 2:**
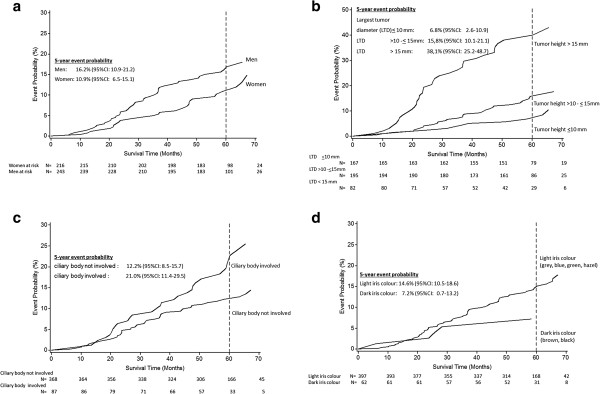
**Estimated probabilities of uveal melanoma death. (a)** stratified by gender adjusted for ciliary body involvement and largest basal tumour diameter among 459 uveal melanoma patients, Germany, 2002–2010**, (b)** stratified by largest basal tumour diameter adjusted for ciliary body involvement among 459 uveal melanoma patients, Germany, 2002–2010, **(c)** stratified by ciliary body involvement adjusted for largest basal tumour diameter among 459 uveal melanoma patients, Germany, 2002–2010, **(d)** stratified by iris colour adjusted for ciliary body involvement and largest basal tumour diameter among 459 uveal melanoma patients, Germany, 2002–2010.

Table 2 presents estimated hazard ratios (HR) of overall mortality and uveal melanoma-specific mortality according to different prognostic factors. We found a 30% decreased HR of death from all causes and from uveal melanoma-specific deaths for women compared to men. After adjustment for ciliary body involvement and basal tumour diameter, the 5-year probability of uveal melanoma death was 10.9% (95% CI: 6.5-15.1) for women and 16.2% (95% CI: 10.9-21.2) for men (Figure 2a). Increasing basal tumour diameter was associated with an increased HR of uveal melanoma death (Figure 2b). By ciliary body involvement, the adjusted 5-year probability of uveal melanoma death was nearly doubled compared with no ciliary body involvement (21.0% (95% CI: 11.4-29.5) versus 12.2% (95% CI: 8.5-15.7)) (Figure 2c). Patients with light iris colour had an adjusted 2.3-fold increased risk of overall and uveal melanoma-specific death compared to brown or black iris colour (Table 2). The adjusted 5-year probability of uveal melanoma death for patients with light iris colour was 14.6% (95% CI: 10.5-18.6), and for patients with dark iris colour 7.2% (95% CI: 0.7-13.2), respectively (Figure 2d). The adjusted HR for women with children was 0.6 (95% CI: 0.2 - 1.7) compared to women without children. An increasing number of children did not lead to a decrease in HR estimates.

**Table 2:** Estimated hazard ratios of overall mortality and melanoma-specific mortality by patient characteristics

	**Patients**		**Number of death**	**Overall mortality N = 95**				**Melanoma-specific mortality N = 79**			
	**N**	**%**	**N**	**HR**^**1)**^	**(95% CI)**^**2)**^	**HR**^**3)**^	**(95% CI)**	**HR**^**1)**^	**(95% CI)**	**HR**^**3)**^	**(95% CI)**
**Gender**											
Men	216	47.1	60	1.0		1.0 ^**7)**^		1.0		1.0 ^**7)**^	
**Women**	243	52.9	35	0.6	(0.4 - 0.9)	0.7	(0.4 - 1.0)	0.6	(0.4-1.0)	0.7	(0.4 - 1.1)
**Largest basal tumour diameter (mm)**											
≤ 10	168	36.6	21	1.0		1.0 ^**8)**^		1.0		1.0 ^**8)**^	
> 10 - ≤15	197	42.9	34	1.4	(0.8 - 2.5)	1.3	(0.8 - 2.3)	2.2	(1.1 - 4.3)	2.0	(1.0 - 4.0)
> 15	79	17.2	38	5.1	(3.0 - 8.8)	4.2	(2.4 - 7.4)	8.4	(4.2 – 16.1)	7.0	(3.5 - 13.9 )
**Extra ocular growth**											
No	451	98.3		1.0		1.0 ^**8)**^		1.0		1.0 ^**8)**^	
Yes	7	1.5	2	1.7	(0.4 - 7.0)	1.2	(0.3 - 4.8)	2.0	(0.5 - 8.2)	1.2	(0.3 - 4.8)
**Ciliary body involvement**											
No	368	80.2	60	1.0		1.0 ^**9)**^		1.0		1.0 ^**9)**^	
Yes	87	19.0	34	2.7	(1.8 - 4.2)	1.8	( 1.1 - 2.8)	3.0	(1.9 - 4.7)	1.7	(1.0 - 2.8)
**Iris colour**											
Dark^4)^	62	13.5	7	1.0		1.0 ^**7)**^		1.0		1.0 ^**7)**^	
Light^5)^	397	86.5	88	2.1	(1.0 - 4.5)	2.0	(0.9 - 4.3)	2.4	(1.0-6.0)	2.3	(0.9 - 5.8)
**Children**^**6)**^											
No	28	13.0	7	1.0		1.0 ^**10)**^		1.0		1.0 ^**10)**^	
Yes	188	87.0	28	0.5	(0.2 - 1.2)	0.4	(0.2 - 1.0)	0.7	(0.3 - 1.7)	0.6	(0.2 - 1.7)
Number of children											
0	28	12.9	7	1.0		1.0 ^**8)**^		1.0		1.0 ^**8)**^	
1	62	28.7	10	0.6	(0.2 - 1.6)	0.5	(0.2 - 1.4)	0.8	(0.3 - 2.3)	0.7	(0.2 - 2.3)
2	87	40.3	12	0.5	(0.2 - 1.3)	0.4	(0.2 - 1.1)	0.5	(0.2 - 1.6)	0.5	(0.2 - 1.5)
≥3	39	18.1	6	0.6	(0.2 - 1.6)	0.4	(0.1 - 1.3)	0.8	(0.2 - 2.5)	0.7	(0.2 - 2.5)

^1)^crude Hazard Ratio ^2)^95% Confidence Interval ^3)^adjusted Hazard Ratio.

^4)^brown, black iris colour ^5)^grey, blue, green, hazel iris colour.

^6)^only women.

^7)^adjusted for ciliary body involvement and largest basal tumour diameter ^8)^adjusted for ciliary body involvement ^9)^adjusted for largest basal tumour diameter ^10)^adjusted for age and graduation.

The value of the bootstrap-corrected C statistics was 0.76 (95% CI: 0.74-0.77), indicating a satisfactory prediction performance of the model.

## Discussion

In our study, we observed a 5-year overall survival of 79.8% and a uveal melanoma-specific survival of 82.9%. Men had a higher overall- and uveal melanoma-specific mortality than women [6]. Damato et al. found that uveal melanomas tend to be larger and more posterior in men than in women [6]. Another reason for the higher mortality in men could be that women usually contact their physician earlier when a problem occurs. Thus a treatment can be initiated before the situation has evolved too dramatically, resulting in better treatment outcome [24]. Our survival rates are comparable with other studies [5,15,25-27]. However, the reported survival rates are difficult to compare because of differences in the corresponding populations of uveal melanoma patients (differences in age, tumour characteristics, and tumour staging). As our and other studies have shown, large tumour diameters and ciliary body involvement are established prognostic factors for uveal melanoma-specific mortality [3,5,16,28,29]. Patients in our study with the largest tumour diameter of greater than 15 millimetres had a nearly 7-fold increased risk of uveal melanoma death compared to patients with a largest tumour diameter smaller than 10 millimetres. We also identified ciliary body involvement to be an independent clinical prognostic factor. The increased 5-year mortality of 21% in patients with ciliary body involvement observed in our study is in line with that reported in other studies [6,16,28]. In large uveal melanomas, the ciliary body is more likely to be involved [30]. Furthermore, they are more likely to show adverse histological and genetic risk factors [10]. Rummel et al. concluded that the poor prognosis for ciliary body involvement might partly be explained by the tendency for these tumours to have microvascular networks and loops, which are associated with uveal melanoma death [31].

We found an association between iris colour and uveal melanoma death. Patients with light iris colour had a 2.3-fold increased risk of death from uveal melanoma compared to patients with dark iris colour, independent of other prognostic factors. This was in line with the study by Regan et al., which was the only study that examined the prognostic influence of iris colour on uveal melanoma death [14]. Among others, they found that the tumours of patients with light iris colour were closer to the optic disc and macular, which is the region of the choroid that is most directly exposed to ultraviolet (UV) radiation [14]. Some case–control studies, including our own study on risk factors associated with uveal melanoma, reported a positive association between UV radiation and uveal melanoma risk [11,13]. Our case–control study found synergistic effects between light iris colour and UV radiation. This interaction analysis suggested that there is an aetiological synergism between light iris colour and exposure to UV radiation [13]. However, the role that light iris colour plays in the metastatic spread of this tumour is unclear. Regan et al. concluded that specific genetic lesions of uveal melanocytes resulting from UV radiation promote uveal melanoma, and this is associated with a more aggressive form of the disease [14].

In addition, our results indicate that women with children have a reduced risk of uveal melanoma death. Our findings are in line with the results from Egan et al.; however, our estimations were not precise [15]. An explanation for this protective effect could be a potential mechanism by which women are “naturally immunised” against cancer antigens by antigens from their foetus. Evidence from recent clinical studies has shown that a high percentage of parous women, but not nulliparous women, showed evidence of immunisation against antigens found against breast, ovarian and endometrial cancer [32].

The usefulness of the risk factors, if collected in a single score, for individual risk prediction was moderate. It is well known that prediction models based on risk factors, even when strongly associated with disease or outcome, are not good predictors for an individual patient [33].

The strengths of our study are the nearly complete follow-up of our patients (99.5%), and survival estimates based on data collected from multiple sources, including death certificates, patients’ ophthalmologists and/or general physicians, as well as from the Division of Ophthalmology, University of Essen, where the patients were treated.

Weaknesses include the fact that some benign lesions, such as choroidal naevi, may have been classified as uveal melanomas, resulting in an overestimation of survival from uveal melanoma. Another limitation is related to the analysis regarding children as a prognostic factor: we asked the patients “do you have children” and not “do you have biological children” which would have been more precise. This, in turn, could lead to a slight overestimation of the number of children. Furthermore, this is a secondary analysis from an original case–control study designed to examine the associations between radiofrequency radiation and the risk of developing uveal melanoma. [19] For the current project, only participants identified as cases were relevant.

## Conclusion

We summarized, light iris colour appears to be not only a risk factor for uveal melanoma but also a prognostic factor for death due to uveal melanoma. More research is needed to firmly establish this link.

## Abbreviations

CI: Confidence Interval; Fig: Figure; HR: Hazard Ratio; Tab: Table; UV: Ultraviolet radiation.

## Competing interests

The authors declare that they have no competing interests.

## Authors’ contributions

ASP conceived of the study, and participated in its design and coordination, performed the statistical analysis, and helped to draft the manuscript. SK participated in the design of the study and performed the statistical analysis. OK made substantial contributions to analysis and interpretation of data. KHJ give final approval of the version to be published. NB made acquisition of data. AS participated in the design of the study and give final approval of the version to be published. All authors read and approved the final manuscript.

## Pre-publication history

The pre-publication history for this paper can be accessed here:

http://www.biomedcentral.com/1471-2415/14/42/prepub
